# Supplementary material for: Flexible comparison of batch correction methods for single-cell RNA-seq using BatchBench
Source: Nucleic Acids Res. 2021 Feb 1;49(7):e42. doi: 10.1093/nar/gkab004 (PMC8053088; doi:10.1093/nar/gkab004)
Supplement: gkab004_Supplemental_File [file gkab004_supplemental_file.pdf]

Supplementary Materials

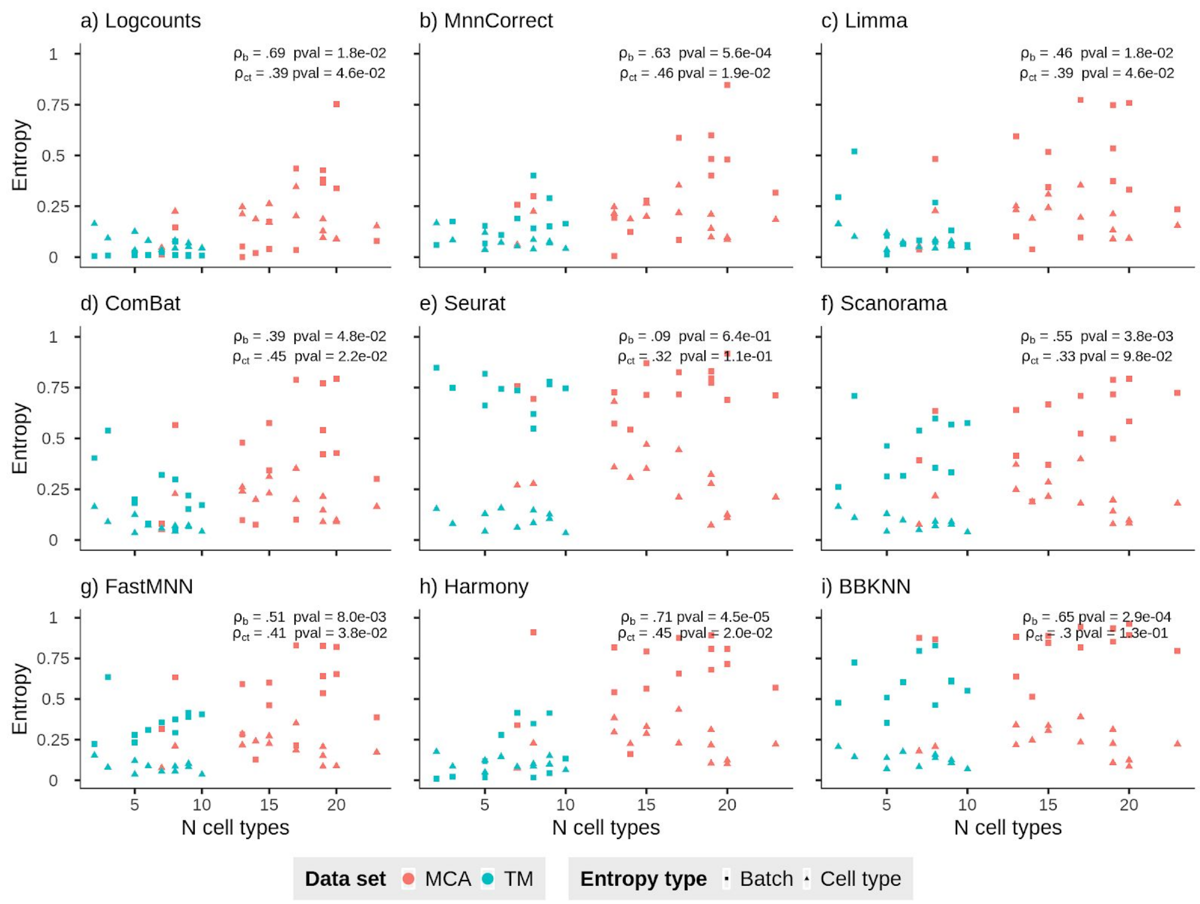

Figure S1. Batch and cell type entropies for eight batch correction methods and three datasets as a function of the number of cell types. The inset text for each panel shows the Spearman's rank correlation coefficient between the number of cell types and batch (ρ_b), or cell type (ρ_{ct}) entropy values.

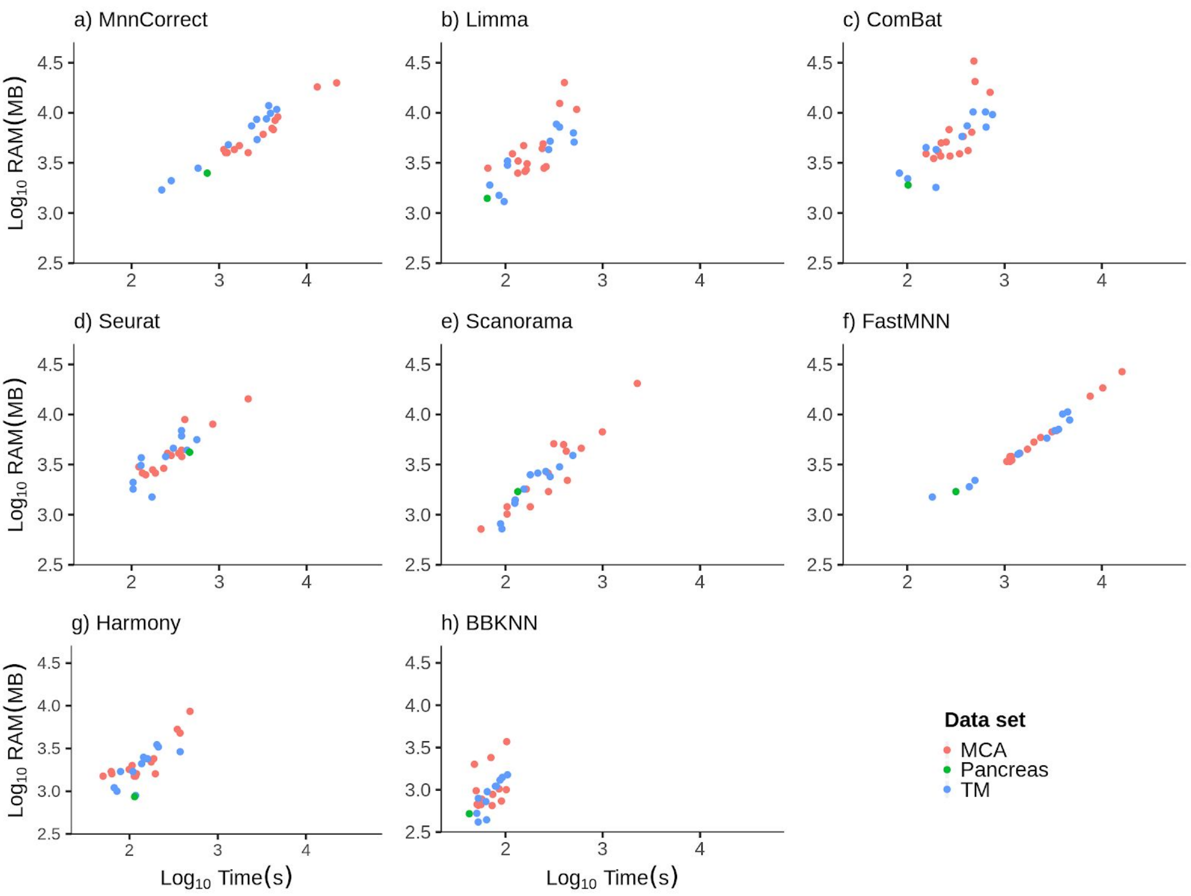

Figure S2. Memory requirements and runtimes for all datasets per method. Values are displayed on the log-10 scale.

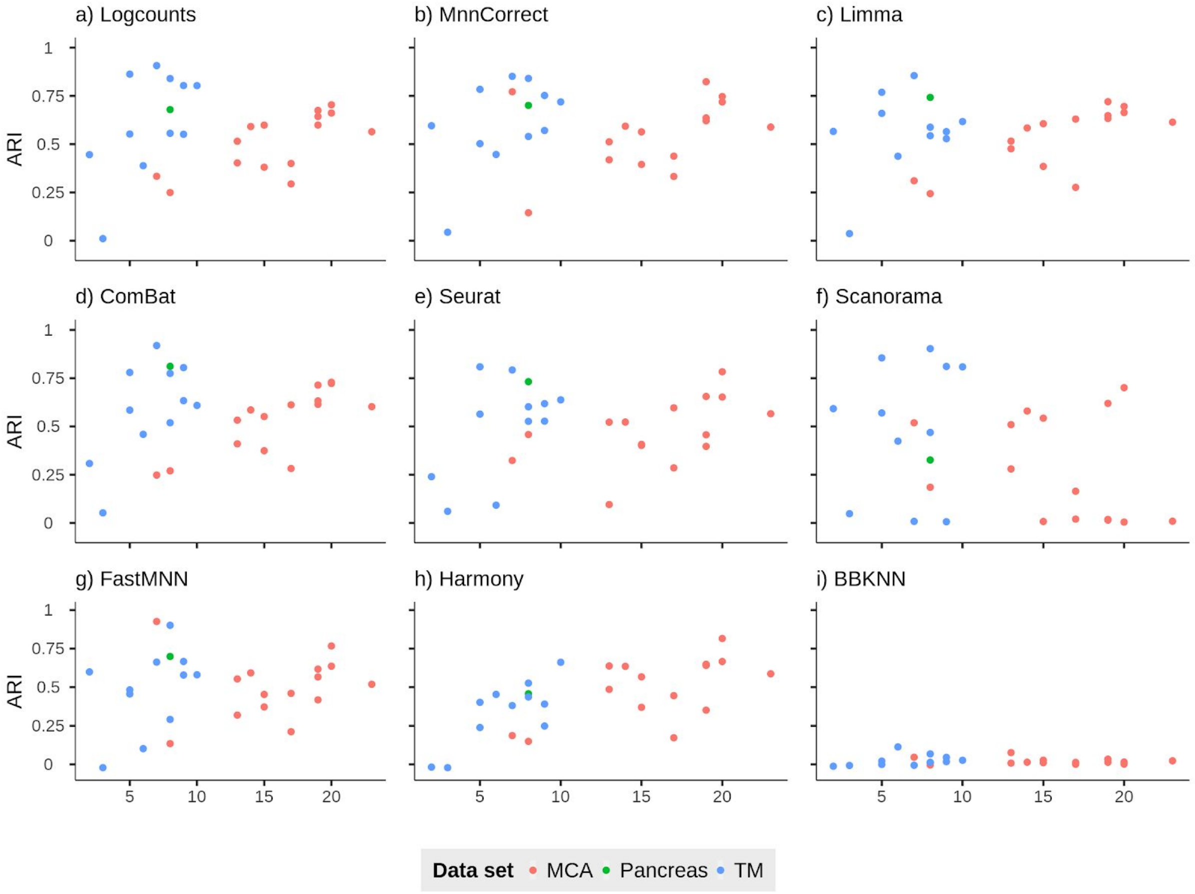

Figure S3. Adjusted Rand Index for the standard hierarchical clustering algorithm as a function of the number of cell types of the datasets.

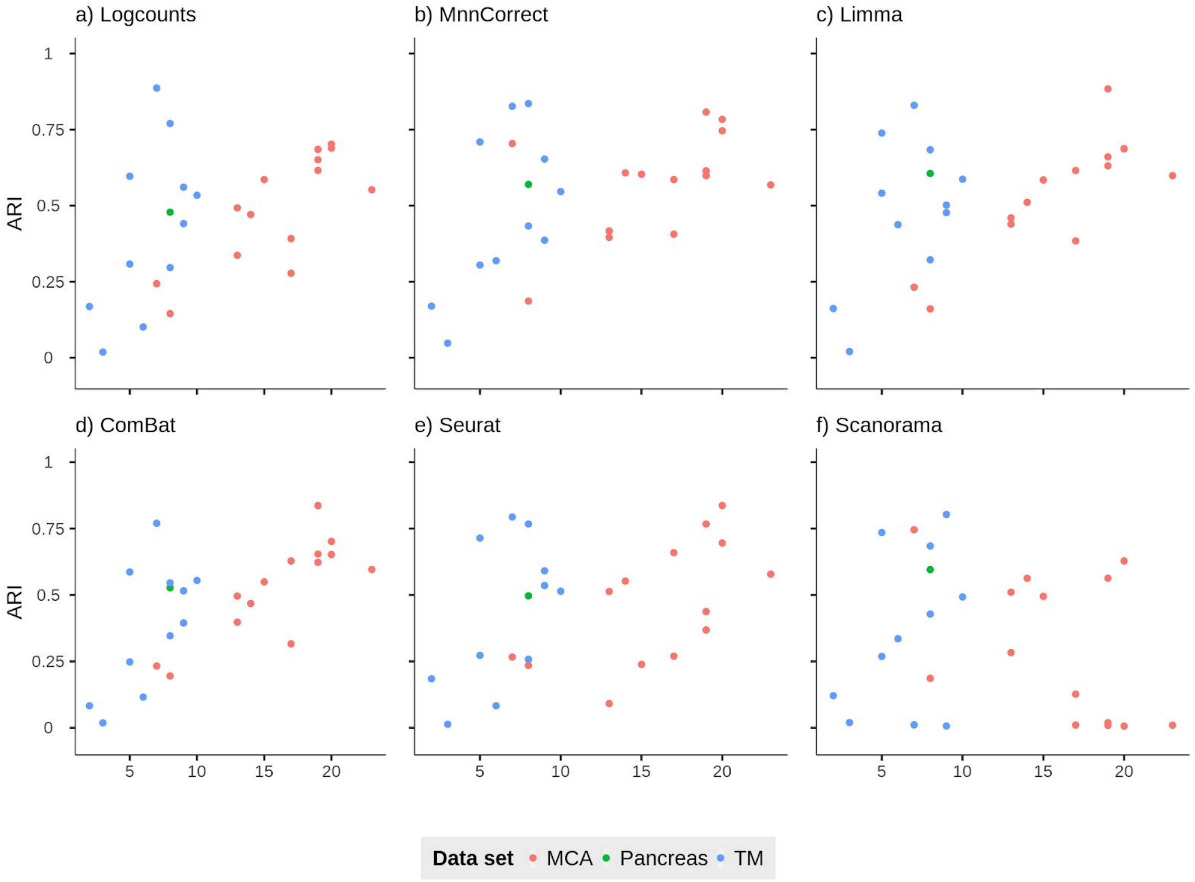

Figure S4. Adjusted Rand Index for the RaceID-hclust clustering algorithm as a function of the number of cell types of the datasets.

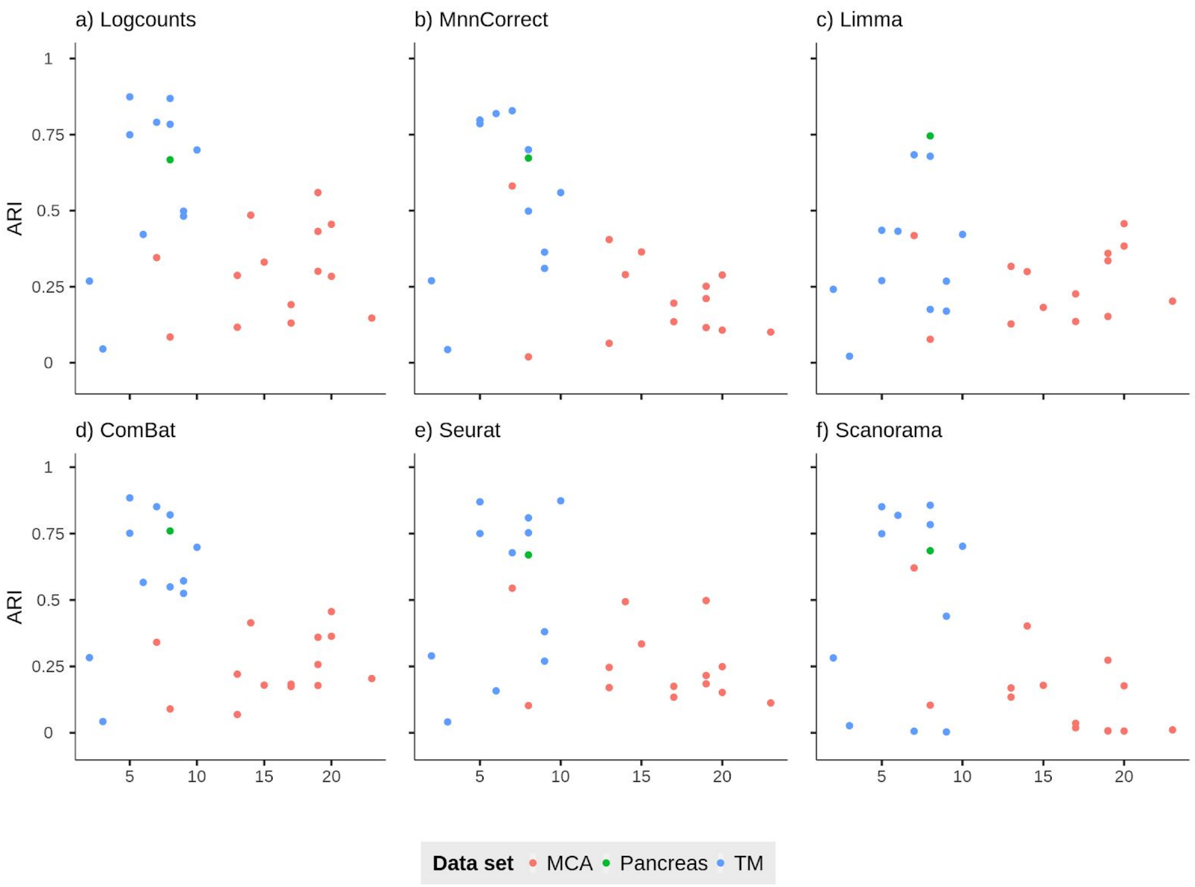

Figure S5. Adjusted Rand Index for the RaceID-kmeans clustering algorithm as a function of the number of cell types of the datasets.

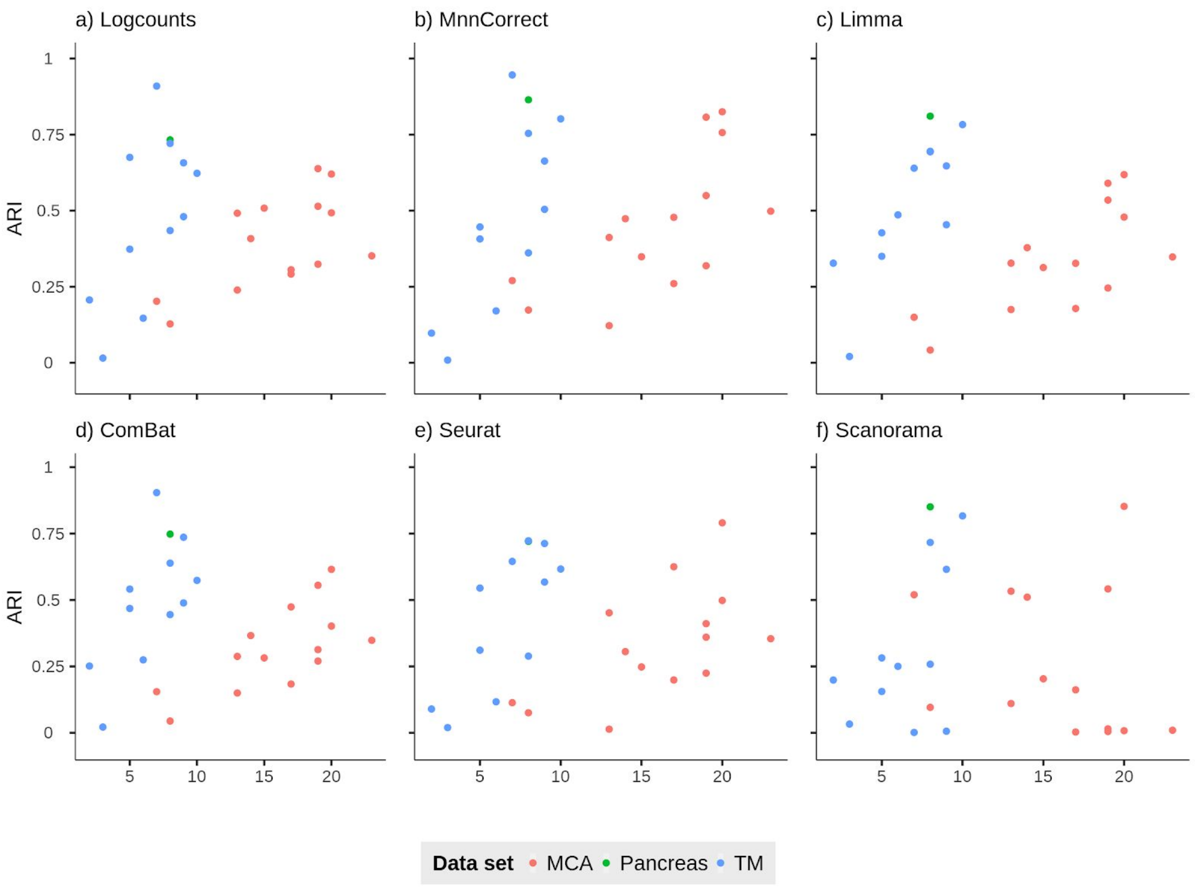

Figure S6. Adjusted Rand Index for the RaceID-kmedoids clustering algorithm as a function of the number of cell types of the datasets.

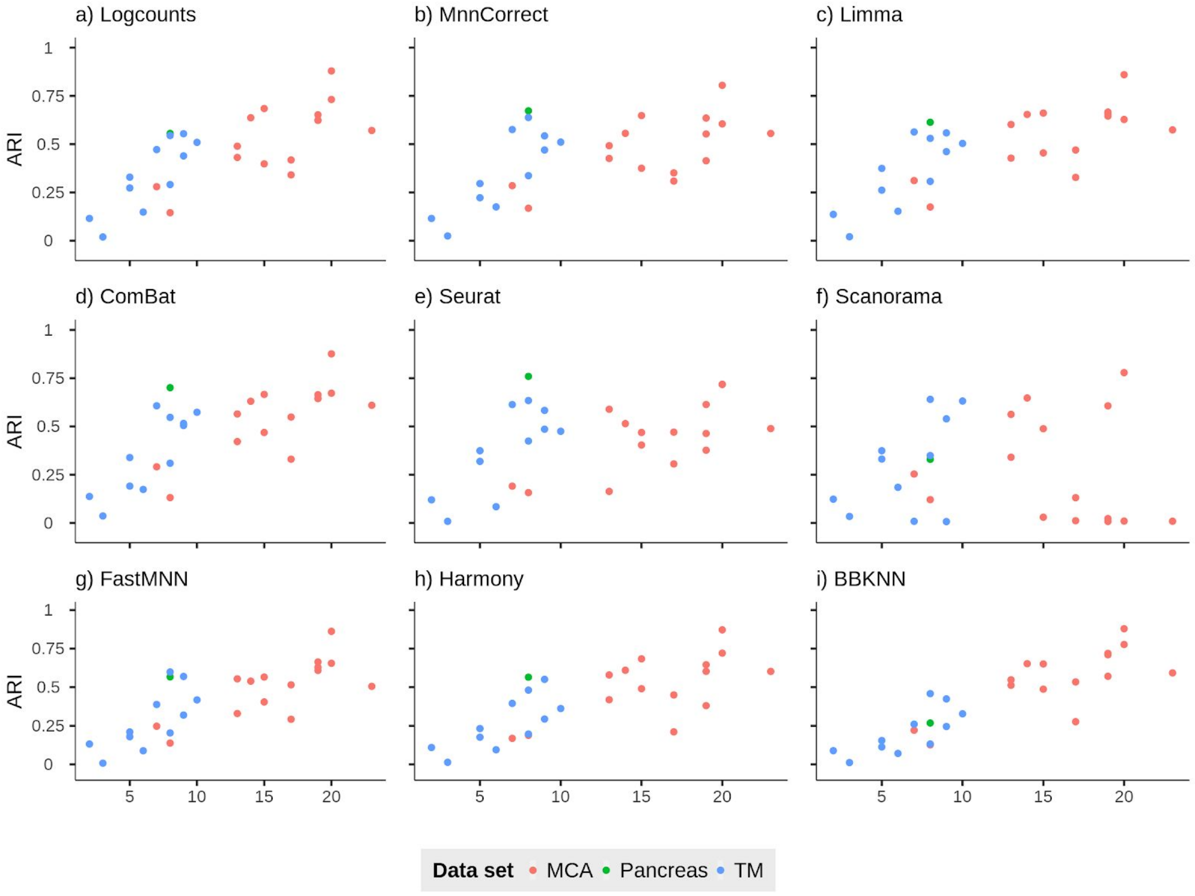

Figure S7. Adjusted Rand Index for the Leiden clustering algorithm as a function of the number of cell types of the datasets.

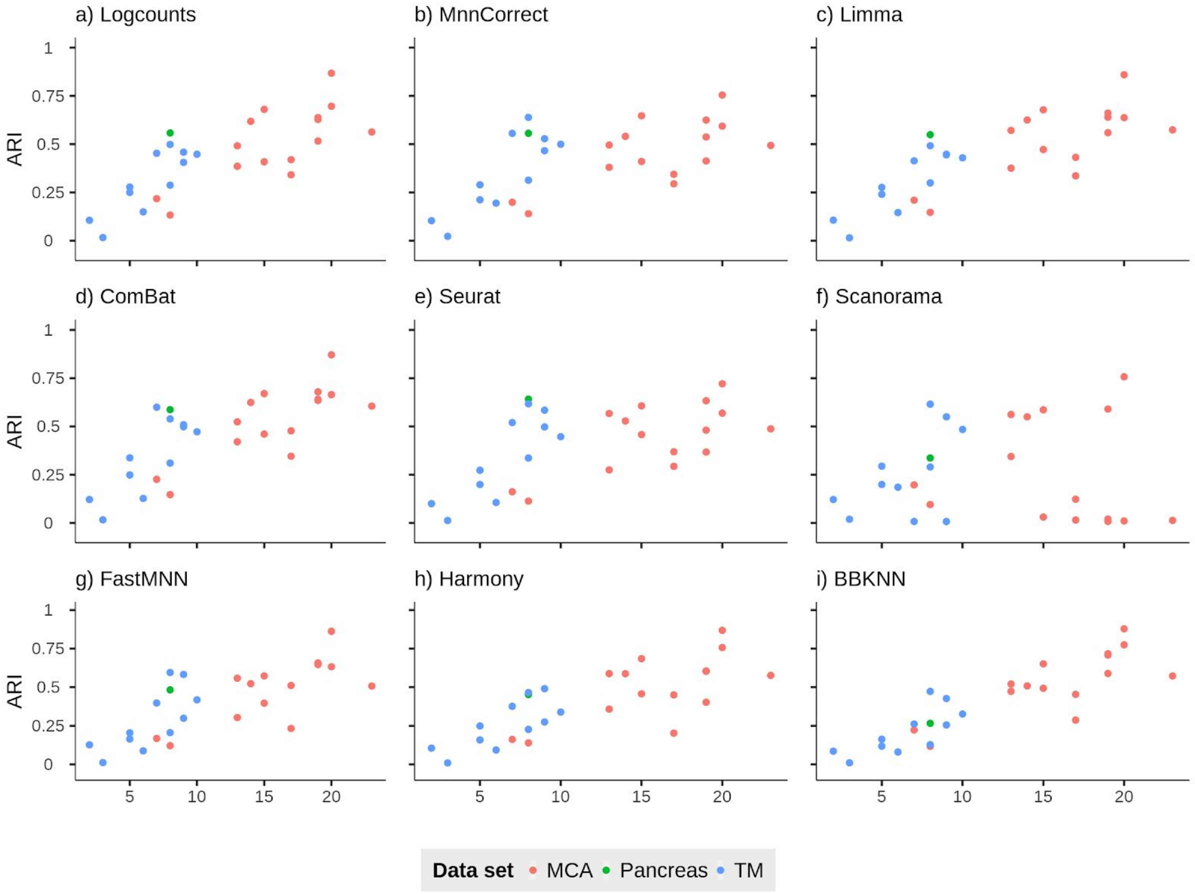

Figure S8. Adjusted Rand Index for the Louvain clustering algorithm as a function of the number of cell types of the datasets.

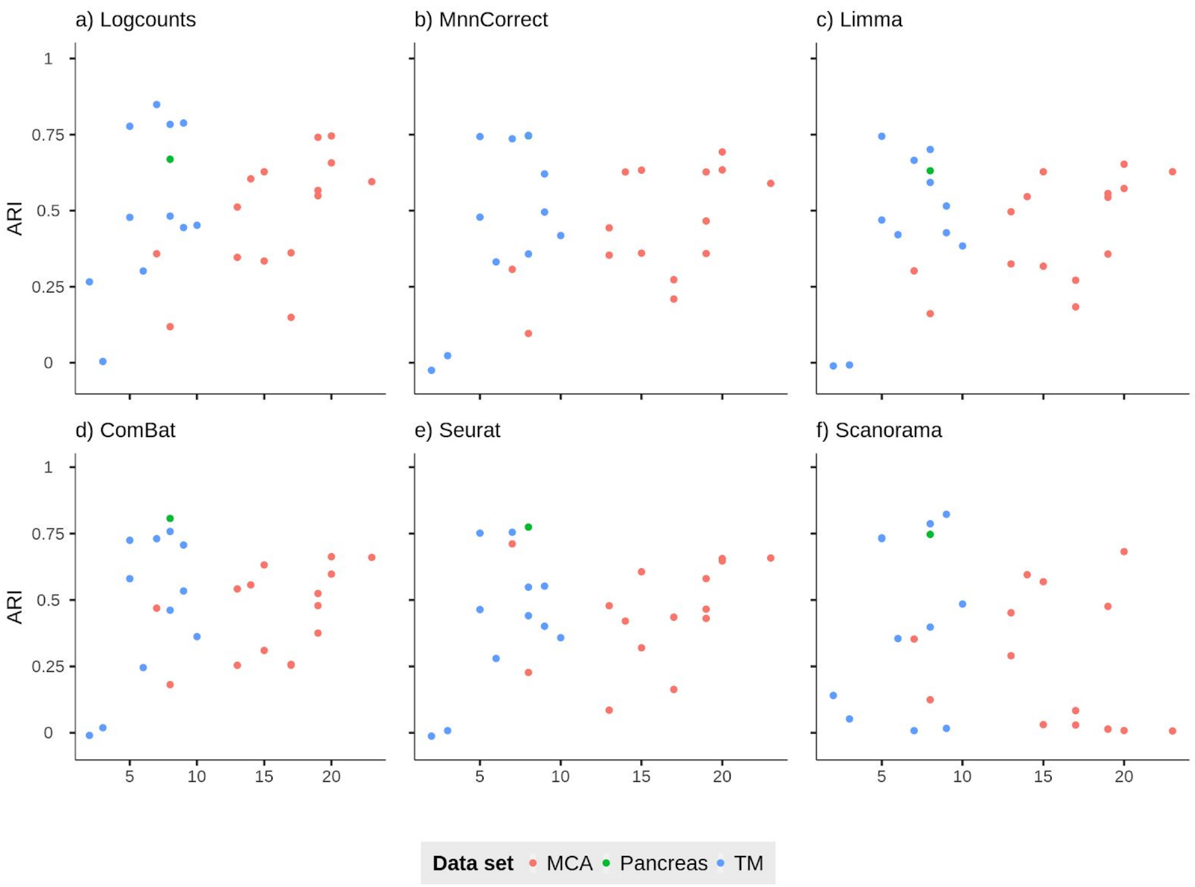

Figure S9. Adjusted Rand Index for the SC3 clustering algorithm as a function of the number of cell types of the datasets.

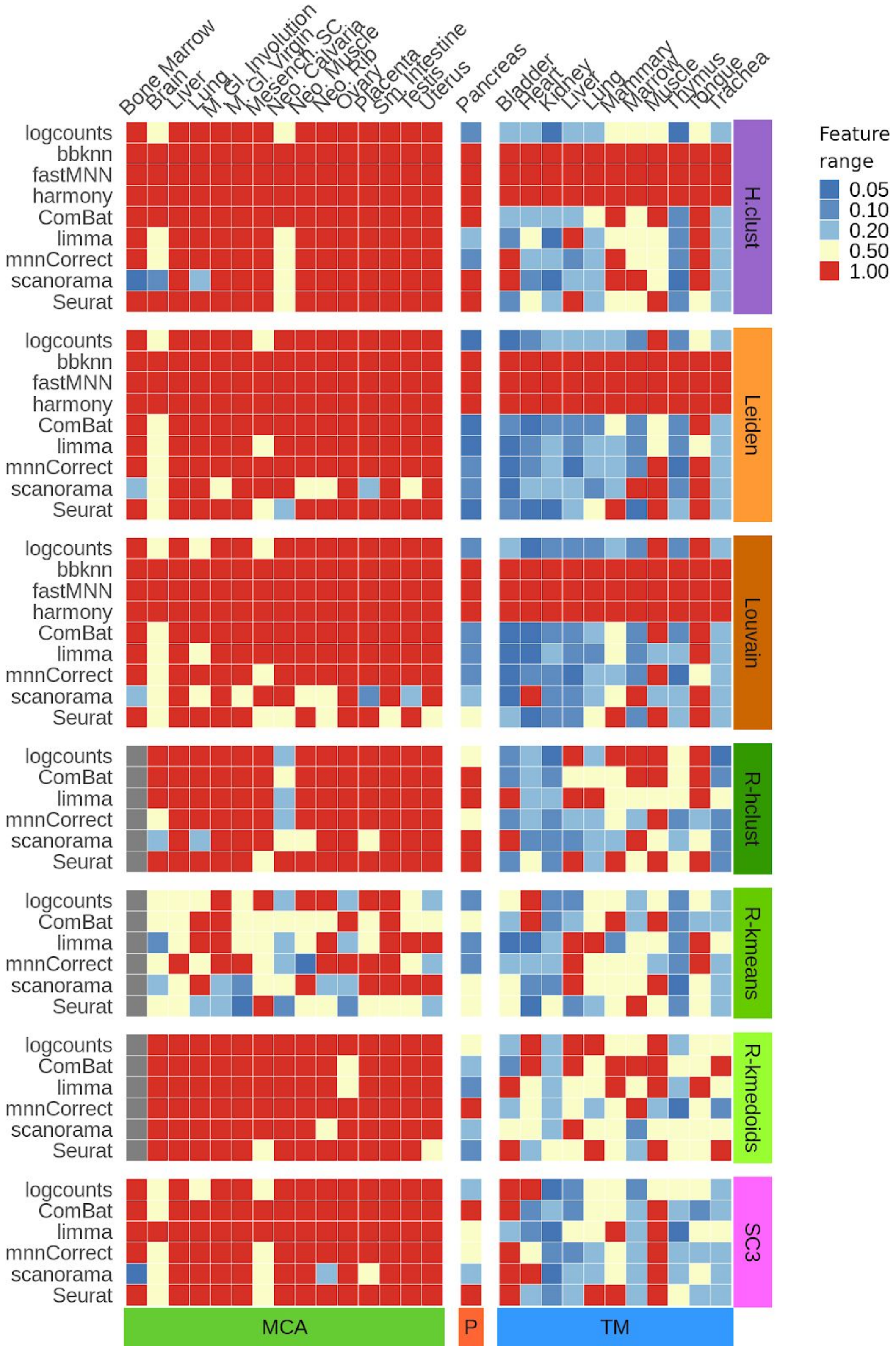

Figure S10. Fraction of features used in clustering analysis for which the highest Adjusted Rand Index is obtained. Methods BBKNN, Harmony and fastMNN are not included since they do not operate in the gene space, and hence no subsetting is performed.

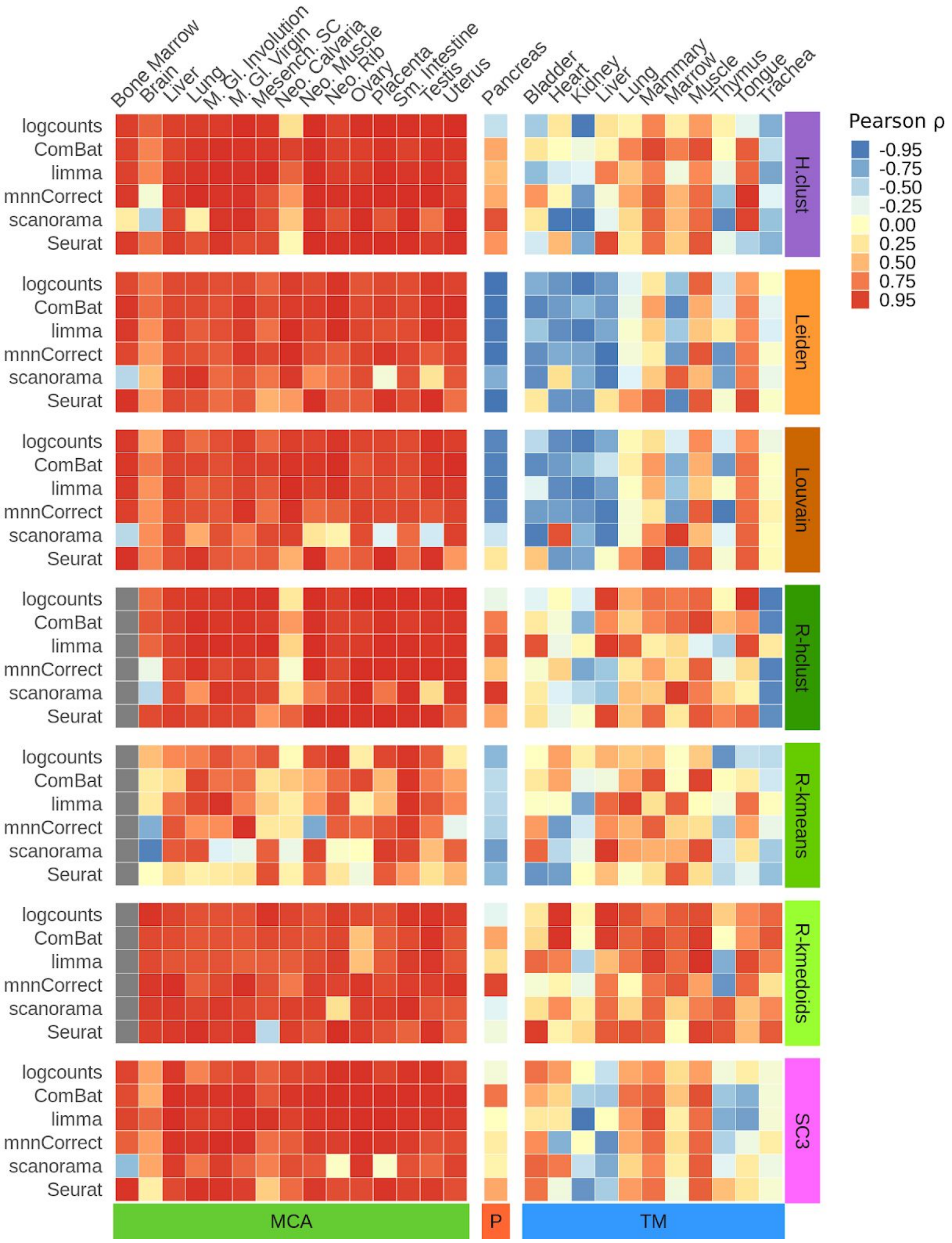

Figure S11. Pearson correlation coefficient between Adjusted Rand Index Values and their corresponding feature fraction used in clustering analysis. Methods BBKNN, Harmony and fastMNN are not included since they do not operate in the gene space, and hence no correlation can be computed.

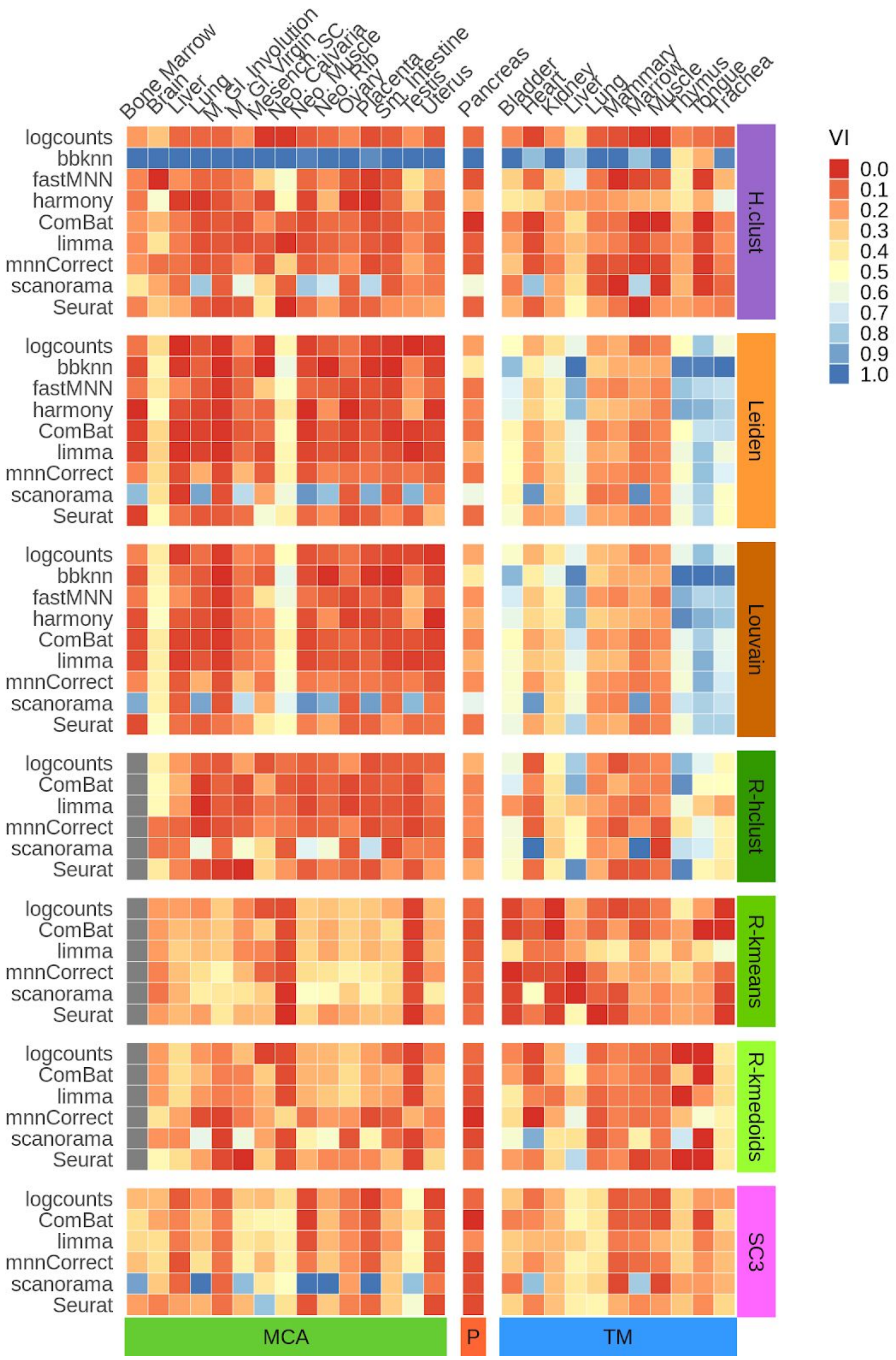

Figure S12. Clustering similarity of batch corrected output to cell labels as evaluated by Variation of Information (VI) distance. The highest VI value from the five fractions of features considered for the clustering is displayed.

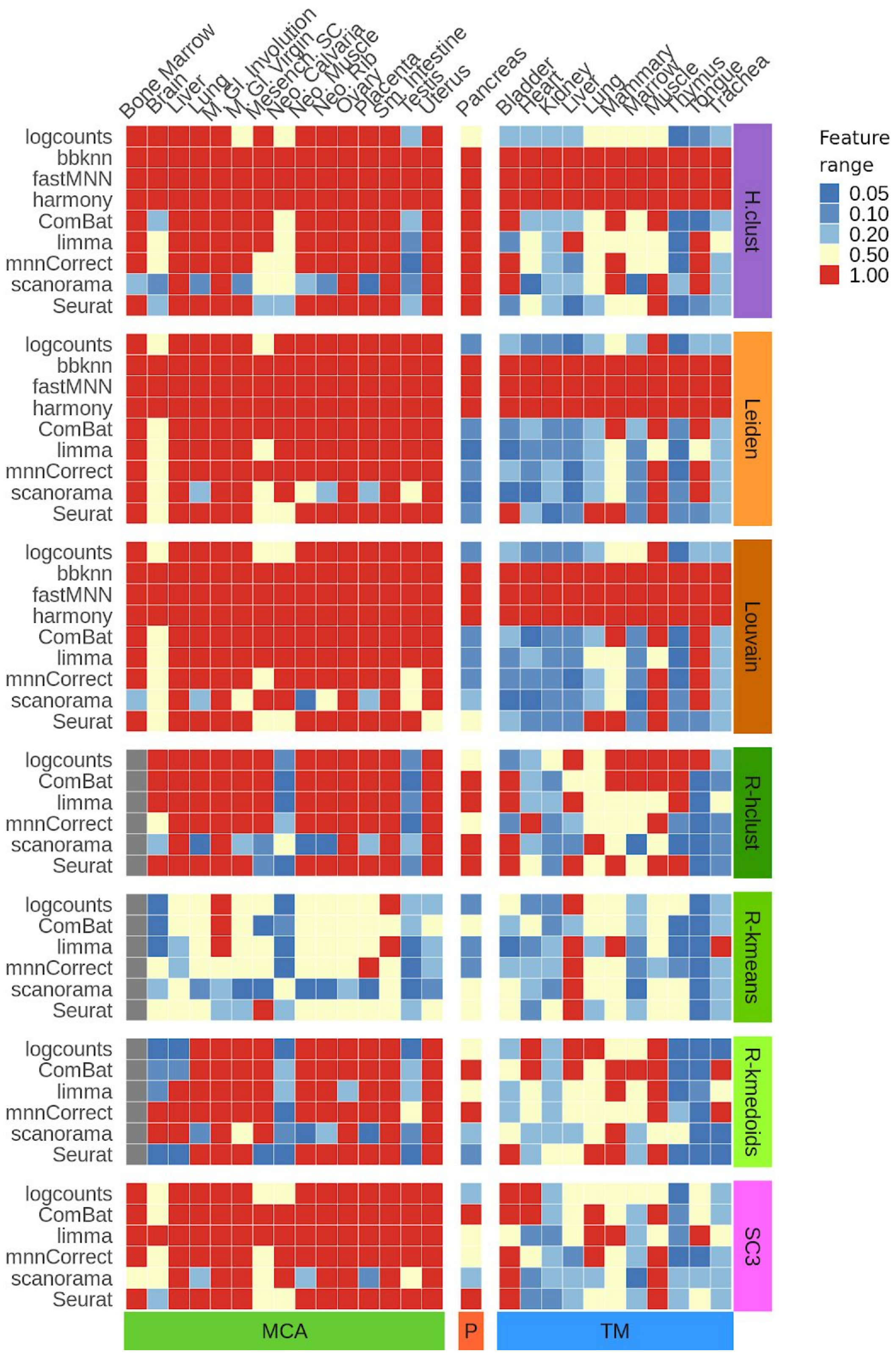

Figure S13. Fraction of features used in clustering analysis for which we obtain the best Variation of Information. Methods BBKNN, Harmony and fastMNN are not included since they do not operate in the gene space, and hence no subsetting is performed.

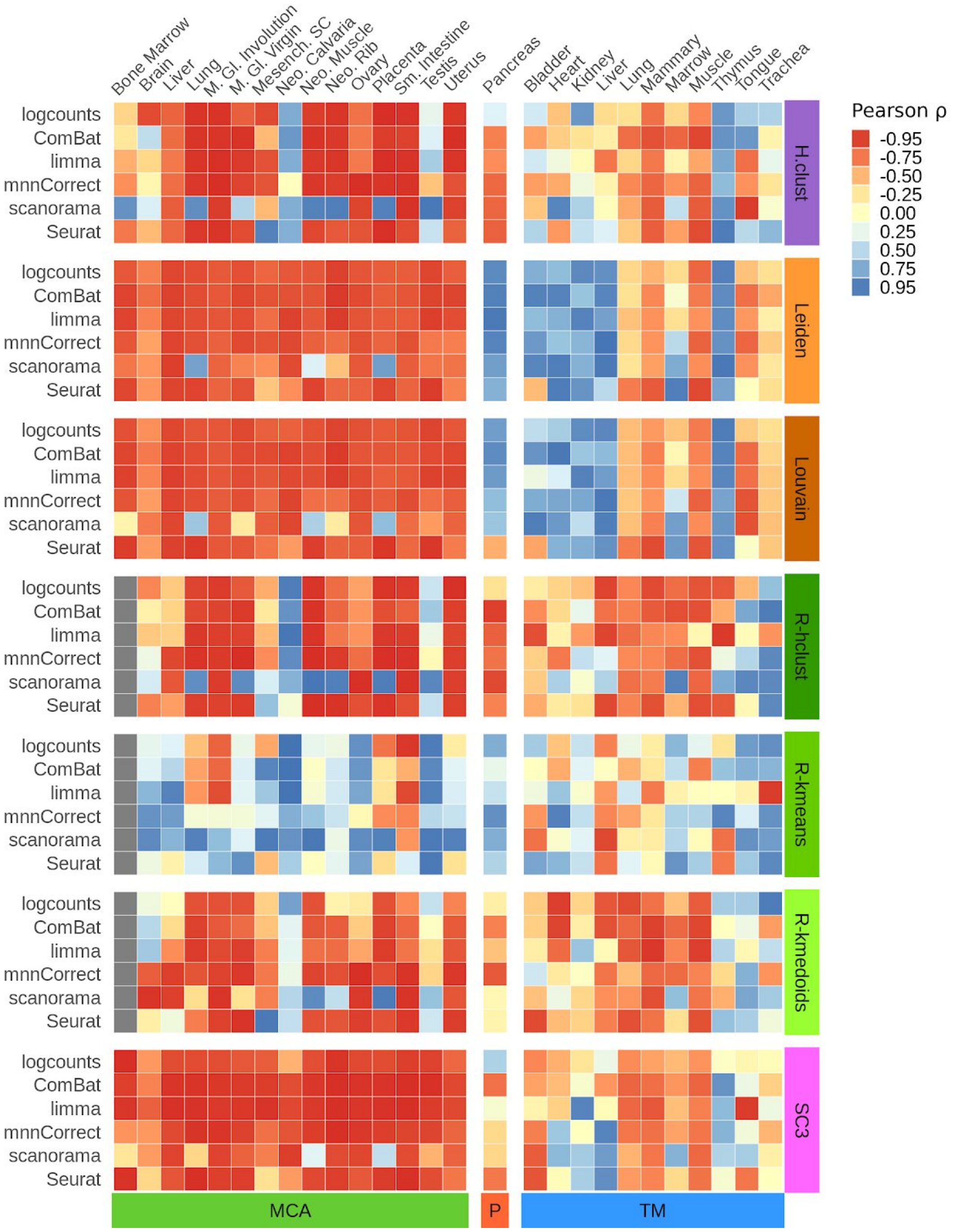

Figure S14. Pearson correlation coefficient between Adjusted Rand Index Values and their corresponding feature fraction used in clustering analysis. Methods BBKNN, Harmony and fastMNN are not included since they do not operate in the gene space, and hence no correlation can be computed.

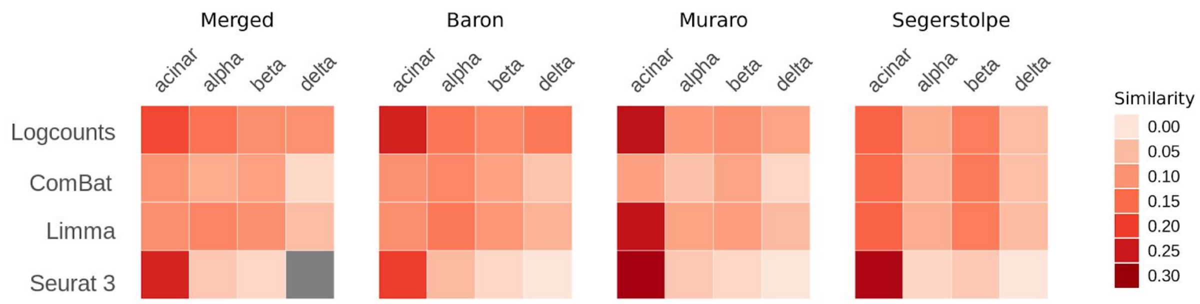

Figure S15. Jaccard similarity index computed between markers from the Pancreas dataset and pancreas marker genes from the CellMarker database.

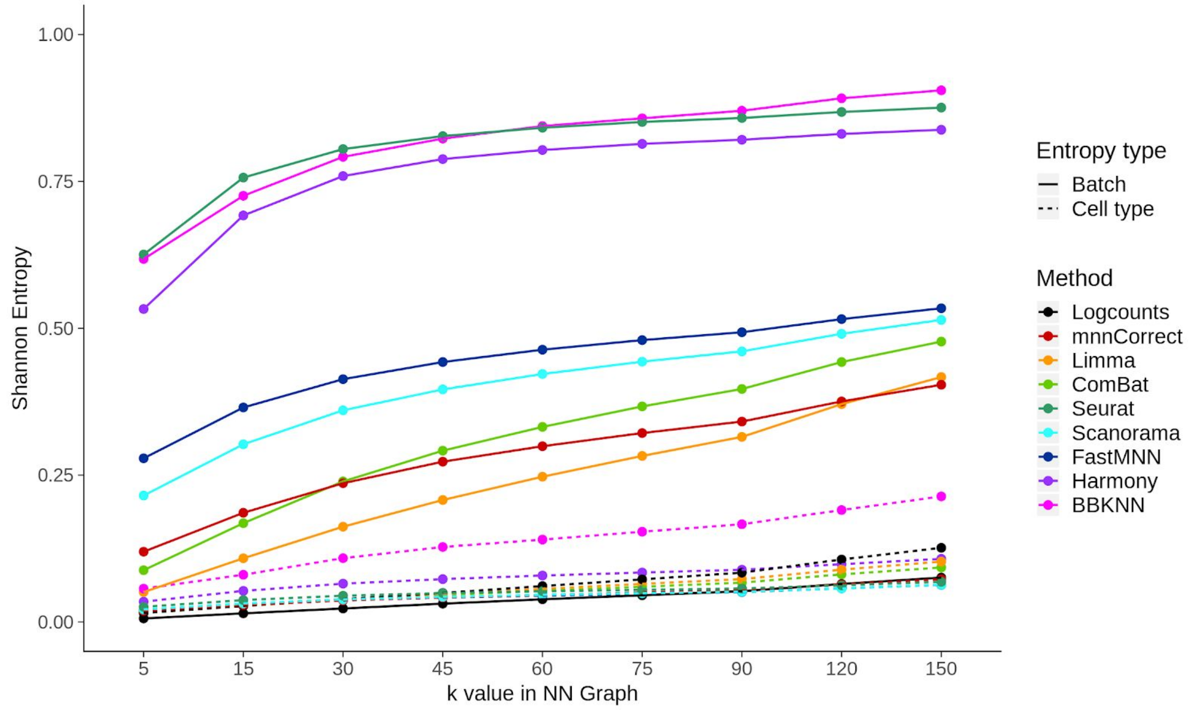

Figure S16. Batch and cell type entropies for the Pancreas dataset using different values of k when building the k-nearest neighbour graph.

N	Data set	Organ	Entropy type	Log-counts	mnn-Correct	Limma	Com-Bat	Seurat	Scano-rama	fast-MNN	Har-mony
1	MCA	Bone Marrow	Batch	0.172	0.27	0.517	0.576	0.87	0.667	0.601	0.793
2	MCA	Bone Marrow	Cell type	0.262	0.265	0.308	0.312	0.47	0.284	0.272	0.33
3	MCA	Brain	Batch	0.011	0.257	0.038	0.08	0.757	0.393	0.316	0.339
4	MCA	Brain	Cell type	0.046	0.061	0.055	0.049	0.269	0.076	0.076	0.075
5	MCA	Liver	Batch	0.02	0.125	0.039	0.076	0.544	0.19	0.126	0.161
6	MCA	Liver	Cell type	0.186	0.187	0.19	0.199	0.307	0.188	0.241	0.224
7	MCA	Lung	Batch	0.427	0.598	0.748	0.771	0.797	0.788	0.827	0.892
8	MCA	Lung	Cell type	0.095	0.097	0.088	0.089	0.073	0.078	0.085	0.104
9	MCA	M. Gl. Involution	Batch	0.752	0.846	0.759	0.794	0.917	0.794	0.819	0.809
10	MCA	M. Gl. Involution	Cell type	0.089	0.087	0.09	0.09	0.109	0.083	0.087	0.101
11	MCA	M. Gl. Virgin	Batch	0.437	0.587	0.774	0.787	0.825	0.71	0.83	0.877
12	MCA	M. Gl. Virgin	Cell type	0.202	0.218	0.194	0.198	0.211	0.18	0.185	0.228
13	MCA	Mesench. SC	Batch	0	0.004	0.101	0.098	0.574	0.415	0.282	0.541
14	MCA	Mesench. SC	Cell type	0.211	0.215	0.231	0.24	0.68	0.371	0.285	0.384
15	MCA	Neonatal Calvaria	Batch	0.146	0.3	0.482	0.566	0.695	0.634	0.633	0.911
16	MCA	Neonatal Calvaria	Cell type	0.225	0.224	0.227	0.227	0.278	0.217	0.21	0.228
17	MCA	Neonatal Muscle	Batch	0.08	0.317	0.235	0.301	0.711	0.723	0.388	0.57
18	MCA	Neonatal Muscle	Cell type	0.152	0.184	0.155	0.165	0.211	0.18	0.172	0.221
19	MCA	Neonatal Rib	Batch	0.381	0.484	0.534	0.542	0.773	0.717	0.64	0.809
20	MCA	Neonatal Rib	Cell type	0.187	0.21	0.211	0.214	0.322	0.197	0.207	0.31
21	MCA	Ovary	Batch	0.052	0.193	0.595	0.479	0.727	0.639	0.591	0.816
22	MCA	Ovary	Cell type	0.247	0.247	0.25	0.261	0.358	0.249	0.217	0.296
23	MCA	Placenta	Batch	0.338	0.48	0.332	0.428	0.69	0.584	0.655	0.715

24	MCA	Placenta	Cell type	0.089	0.097	0.093	0.098	0.125	0.097	0.088	0.122
25	MCA	Small Intestine	Batch	0.366	0.402	0.373	0.421	0.83	0.498	0.534	0.679
26	MCA	Small Intestine	Cell type	0.128	0.14	0.132	0.147	0.277	0.141	0.151	0.217
27	MCA	Testis	Batch	0.035	0.084	0.097	0.101	0.716	0.524	0.215	0.656
28	MCA	Testis	Cell type	0.345	0.352	0.353	0.352	0.443	0.398	0.351	0.435
29	MCA	Uterus	Batch	0.04	0.277	0.345	0.343	0.715	0.371	0.461	0.563
30	MCA	Uterus	Cell type	0.171	0.2	0.242	0.23	0.351	0.214	0.225	0.288
31	TM	Bladder	Batch	0.022	0.068	0.012	0.183	0.817	0.313	0.278	0.018
32	TM	Bladder	Cell type	0.126	0.119	0.119	0.125	0.129	0.13	0.119	0.123
33	TM	Heart	Batch	0.021	0.189	0.082	0.321	0.737	0.538	0.355	0.414
34	TM	Heart	Cell type	0.035	0.053	0.049	0.058	0.062	0.05	0.055	0.083
35	TM	Kidney	Batch	0.077	0.4	0.269	0.298	0.548	0.597	0.373	0.349
36	TM	Kidney	Cell type	0.082	0.085	0.083	0.07	0.084	0.091	0.085	0.086
37	TM	Liver	Batch	0.011	0.11	0.065	0.081	0.744	0.316	0.309	0.279
38	TM	Liver	Cell type	0.081	0.071	0.073	0.073	0.157	0.097	0.088	0.145
39	TM	Lung	Batch	0.008	0.165	0.06	0.171	0.745	0.576	0.406	0.133
40	TM	Lung	Cell type	0.045	0.041	0.045	0.041	0.034	0.039	0.036	0.063
41	TM	Mammary	Batch	0.009	0.142	0.074	0.049	0.619	0.356	0.293	0.017
42	TM	Mammary	Cell type	0.043	0.038	0.043	0.043	0.146	0.07	0.055	0.101
43	TM	Marrow	Batch	0.004	0.151	0.059	0.153	0.779	0.334	0.414	0.414
44	TM	Marrow	Cell type	0.051	0.068	0.055	0.064	0.125	0.077	0.103	0.15
45	TM	Muscle	Batch	0.009	0.29	0.131	0.219	0.767	0.568	0.389	0.044
46	TM	Muscle	Cell type	0.068	0.076	0.078	0.071	0.105	0.09	0.083	0.099
47	TM	Thymus	Batch	0.008	0.175	0.52	0.539	0.748	0.71	0.635	0.023
48	TM	Thymus	Cell type	0.093	0.083	0.1	0.089	0.081	0.109	0.08	0.085
49	TM	Tongue	Batch	0.005	0.06	0.294	0.403	0.848	0.261	0.223	0.009
50	TM	Tongue	Cell type	0.164	0.167	0.164	0.164	0.153	0.165	0.154	0.176
51	TM	Trachea	Batch	0.009	0.153	0.104	0.2	0.661	0.463	0.233	0.119
52	TM	Trachea	Cell type	0.034	0.037	0.035	0.035	0.042	0.041	0.037	0.049
53	Panc-reas	Pancreas _1	Batch	0.026	0.282	0.241	0.237	0.819	0.406	0.413	0.759

54	Panc-reas	Pancreas_1	Cell type	0.039	0.038	0.039	0.039	0.05	0.04	0.037	0.065
55	Panc-reas	Pancreas_2	Batch	0.008	0.133	0.143	0.189	0.829	0.257	0.321	0.733
56	Panc-reas	Pancreas_2	Cell type	0.045	0.039	0.044	0.046	0.039	0.039	0.039	0.077
57	Panc-reas	sub_Pancreas_1	Batch	0.038	0.385	0.373	0.298	0.833	0.458	0.478	0.749
58	Panc-reas	sub_Pancreas_1	Cell type	0.041	0.039	0.045	0.049	0.086	0.039	0.047	0.083
59	Panc-reas	sub_Pancreas_2	Batch	0.114	0.584	0.511	0.486	0.755	0.629	0.679	0.756
60	Panc-reas	sub_Pancreas_2	Cell type	0.09	0.057	0.083	0.075	0.054	0.069	0.046	0.076

Table S1. Entropy values per dataset (MCA: Mouse Cell Atlas, TM: Tabula Muris).

N	Data set	Organ	Pre QC				Post QC			
			N cell	N gene	N cell type	N batch	N cell	N gene	N cell type	N batch
1	MCA	Bone Marrow	26993	12855	19	4	25567	11410	15	3
2	MCA	Brain	4038	16906	15	2	3877	8519	7	2
3	MCA	Liver	4685	15491	20	2	4543	7936	14	2
4	MCA	Lung	6940	17097	32	3	6474	10117	20	3
5	MCA	M. Gl. Involution	4821	15060	24	2	4724	7519	20	2
6	MCA	M. Gl. Virgin	5380	13618	18	4	5371	7996	17	4
7	MCA	Mesench. SC	14684	15941	22	2	14062	10922	13	2
8	MCA	Neonatal Calvaria	7964	17779	14	2	7685	8235	8	2
9	MCA	Neonatal Muscle	4873	16685	27	2	4596	7786	23	2
10	MCA	Neonatal Rib	6262	16346	25	3	6013	8496	19	3
11	MCA	Ovary	4363	16643	14	2	4327	9012	13	2
12	MCA	Placenta	4346	17274	28	2	4017	7804	20	2
13	MCA	Small Intestine	6684	15127	28	3	6153	9569	19	3
14	MCA	Testis	14005	22762	19	2	13787	12969	17	2
15	MCA	Uterus	3739	16705	19	2	3635	8960	15	2
16	TM	Bladder	3879	11433	5	2	3879	11433	5	2

17	TM	Heart	5192	8065	10	2	4773	8065	7	2
18	TM	Kidney	2982	5878	8	2	2670	5878	8	2
19	TM	Liver	1734	7658	6	2	1680	7658	6	2
20	TM	Lung	6486	11391	18	2	6163	11391	10	2
21	TM	Mammary	6467	11376	8	2	6345	11376	8	2
22	TM	Marrow	8296	10750	10	2	8089	10750	9	2
23	TM	Muscle	5607	10425	9	2	5533	10425	9	2
24	TM	Thymus	2761	8135	3	2	2726	8135	3	2
25	TM	Tongue	8952	11290	3	2	8893	11290	2	2
26	TM	Trachea	9981	11547	7	2	9350	11547	5	2
27	TM_Atlas	TM_Atlas_QC_1	62337	4168	51	2	4168	60828	51	2
28	Pancreas	Pancreas_1	9687	1898	18	3	9382	1898	8	3
29	Pancreas	Pancreas_2	9687	7098	18	3	9382	7098	8	3
30	Pancreas	sub_Pancreas_1	4843	1898	5	3	4843	1898	5	3
31	Pancreas	sub_Pancreas_2	3000	1898	17	3	2918	1898	9	3

Table S2: Summary statistics for the datasets considered in this study (MCA: Mouse Cell Atlas, TM: Tabula Muris).
